# Naturally Acquired Antibodies against *Plasmodium falciparum*: Friend or Foe?

**DOI:** 10.3390/pathogens10070832

**Published:** 2021-07-02

**Authors:** Muyideen Kolapo Tijani, Allan Lugaajju, Kristina E. M. Persson

**Affiliations:** 1 Cellular Parasitology Programme, Cell Biology and Genetics Unit, Department of Zoology, University of Ibadan, 200284 Ibadan, Oyo State, Nigeria; muyideen_kolapo.tijani@med.lu.se; 2 Department of Laboratory Medicine, Lund University, Skåne University Hospital, 22185 Lund, Sweden; allan.lugaajju@mak.ac.ug; 3 School of Biomedical Sciences, College of Health Sciences, Makerere University, Kampala P.O. Box 7072, Uganda

**Keywords:** malaria, *Plasmodium falciparum*, immunity, antibodies

## Abstract

Antibodies are central to acquired immunity against malaria. *Plasmodium falciparum* elicits antibody responses against many of its protein components, but there is also formation of antibodies against different parts of the red blood cells, in which the parasites spend most of their time. In the absence of a decisive intervention such as a vaccine, people living in malaria endemic regions largely depend on naturally acquired antibodies for protection. However, these antibodies do not confer sterile immunity and the mechanisms of action are still unclear. Most studies have focused on the inhibitory effect of antibodies, but here, we review both the beneficial as well as the potentially harmful roles of naturally acquired antibodies, as well as autoantibodies formed in malaria. We discuss different studies that have sought to understand acquired antibody responses against *P. falciparum* antigens, and potential problems when different antibodies are combined, such as in naturally acquired immunity.

## 1. Introduction

Malaria still inflicts an extreme level of burden on people living in endemic regions [1]. Africa carries the largest share of this burden, which could also be further aggravated in some areas by wars, natural disasters, and epidemics or pandemics of other serious diseases such as Covid-19 [2]. Malaria is caused by different *Plasmodium* species and is transmitted in human populations by bites of infective female Anopheline mosquitoes. The success of *Plasmodium* species as parasitic organisms is based on their ability to evade immune attacks directed against them by the human host, as well as the mosquito vector. During evolution, the genetic background of both humans and mosquitoes has been of importance in forming which parasites can multiply successfully.

Antibodies are considered an efficient product of the immune system and they are generally produced by B cells/plasma cells, but there is an increasing body of evidence to support antibody production by cancerous and normal non-B cells, such as in proximal tubuli cells and epithelial cells [3,4,5,6]. Naturally acquired antibodies against infectious agents can exert their effector functions by simple binding (steric hindrance), complement activation, cellular cytotoxicity, and opsonophagocytosis [7]. The attention of the scientific world was called to the importance of antibodies in malaria immunology by exquisitely performed experiments where plasma obtained from adult or cord blood was used to treat parasitological and clinical symptoms of malaria in sick children [8,9]. Later studies have doubted that antibodies should work as a treatment, but the idea of using monoclonal antibodies as part of a treatment protocol is a new possibility [10], even though this kind of treatment might be available mainly for travelers. Despite the fact that the malaria parasite presents a number of antigens to the immune system, which has the ability to generate a substantial variability in the production of antibodies, most people living in endemic regions are still not able to maintain high levels of effective antibodies for a long period of time. The half-life of antibodies against measles has, for example, been estimated to be around 200 years [11], while antibodies against malaria parasites are only stable for a few months [12,13,14]. The widespread presence of atypical memory B cells in endemic areas could be one of the reasons for the immune inefficiency [15,16], but there is also evidence that this set of B cells participates in the production of parasite neutralizing antibodies [17]. Here, we review the current knowledge about naturally acquired antibodies elicited against *P. falciparum* and highlight antibody interactions with important antigens expressed as the parasite goes through different stages in the human host as well as the mosquito.

## 2. Antibody Response in the Dermis and at the Liver Stage

The complex and somewhat treacherous interaction of malaria parasites with the human host begins when a parasite-infected female *Anopheles* mosquito injects about 10–150 sporozoites into human skin [18]. Recent studies using human skin explant revealed that sporozoites move rapidly through the dermis [19,20] in a similar way as was observed in rodent *Plasmodium,* both in vitro and in vivo [21,22]. Moreover, based on rodent *Plasmodium* studies, there is evidence to show that about half of the inoculated sporozoites could remain in the skin where they form extrahepatic exoerythrocytic forms. *P. falciparum* is not known to induce a significant dermal immune response, but a recent study based on a *Plasmodium berghei* animal model showed that anti-sporozoite antibodies targeting mainly the circumsporozoite protein (CSP) have protective functions by inhibiting sporozoite motility through the skin [23]. Although this antibody-mediated protection against sporozoites at the dermal stage has not been demonstrated in naturally infected individuals, it could represent a new level in our understanding of the versatility of antibody responses to *Plasmodium* species.

The few sporozoites that are able to make it to the bloodstream quickly invade the hepatocytes and are exposed to the immune system for a relatively short period of time. However, naturally acquired antibodies against whole *P. falciparum* sporozoites have been demonstrated in endemic areas [24,25]. Circumsporozoite protein (CSP) is the dominant antigen on sporozoites and contains an amino acid central repeat region that is composed of different numbers of asparagine–alanine–asparagine–proline (NANP) repeats. Strong antibody responses have been found to be produced against NANP repeats [26,27,28,29,30] and against the other regions of CSP [31]. Naturally acquired antibodies against whole sporozoites are more widespread and could even be higher in children [30] than antibodies against the dominant repeat region (NANP) of CSP. This indicates that antibodies are also elicited against other sporozoite antigens than the immunodominant CSP. Functional activities of anti-sporozoite antibodies have not been well characterized, but recent evidence has shown that naturally acquired IgG antibodies directed against CSP can fix the complement to prevent hepatocyte invasion and kill parasites in vitro [32].

## 3. Antibodies against Merozoites

The asexual stage of *P. falciparum* is responsible for all known symptoms of malaria; thus, functional antibodies against parasite antigens at this stage should be able to reduce or prevent clinical symptoms of malaria. Merozoite-filled vesicles, merozomes, from parasite-infected hepatocytes release merozoites into the blood stream where they invade erythrocytes to begin a 48-h life cycle that will lead to an exponential increase in production of merozoites. Merozoites in the bloodstream are more exposed to the immune system and studies have identified naturally produced antibodies against a majority of the known merozoite surface proteins as well as against merozoite organelles such as rhoptries and micronemes [33].

Merozoite surface protein 1 (MSP1) is the most abundant protein on the merozoites and plays an important role in erythrocyte binding and invasion [34]. Some studies have found correlations between anti-MSP1 antibodies and protection against clinical symptoms, while others have not [33,35]. Anti-MSP1 antibodies have been shown to inhibit erythrocyte invasion, enhance monocyte-mediated phagocytosis of parasites, and aid in complement fixation [36,37,38]. Vaccination of humans with full-length MSP1 leads to the production of antibodies with several of these effector mechanisms [39]. MSP1 is produced as a 196-kDa precursor that undergoes a two-step proteolytic processing essential for both egress [40] and invasion. There is evidence for the existence of naturally acquired antibodies that block this important proteolytic processing of MSP1 [41], called processing inhibitory antibodies, but it is interesting to note that the same study also found antibodies that could interrupt the binding of the processing inhibitory antibodies. Thus, any protective advantage that may be associated with interrupting the MSP1 proteolytic process could also be abrogated by these blocking antibodies, because they target the same epitope. This is one of the few studies that have elegantly shown that antibodies can be beneficial not only for the human, but also for the parasite. Binding of blocking antibodies to the epitope in the presence of processing inhibitory antibodies would ensure the parasite full proteolytic processing of MSP1 and thus a proper invasion of RBCs. Most studies in the field of malaria have focused on finding the antibodies that inhibit the parasites, since these antigens could be potential vaccine targets, but we should probably put more emphasis on showing those results that could also be beneficial for the parasites. Having antibodies that can be both good and bad could be one of the explanations for why no malaria vaccine has yet proven to be fully successful. We have ourselves, for example, shown that inhibitory results can be severely affected depending on which parasite line of *P. falciparum* is used for the experiments [42]. When purified plasma samples (containing mostly antibodies) from endemic areas of Tanzania were used in growth inhibitory assays, parasite-specific antibodies in most samples inhibited the growth of 3D7 and K1 *P. falciparum* lines, but when W2mef was used, the growth was actually enhanced, sometimes as much as 25–50%. It is well established that in endemic areas, many different parasites circulate [43,44] and if antibodies are produced naturally that can enhance the growth of parasites, this is an efficient way for parasites to avoid getting cleared by the human immune system and this should be studied more in detail. It is also known that parasites can vary their invasion pathways to evade inhibitory antibodies [45] and there is evidence that an increase in the growth of parasites can be obtained when naturally acquired antibodies from some individuals are added to *P. falciparum* parasites in vitro [46]. For other pathogens such as the parasite *Leishmania* or viruses like Zika and Dengue, it has been shown that antibody-mediated responses involving the complement or FcR pathways do not always lead to protection or reduced infection, but can sometimes be exploited by these pathogens for enhanced invasion of host cells [47]. This phenomenon is known as antibody-dependent enhancement (ADE) and it is not clear whether *P. falciparum* could also use this mechanism to enhance hepatocyte or erythrocyte invasion. A study suggested that this could definitely be possible, since a monoclonal antibody obtained from MSP1_42_ vaccines enhanced parasite invasion and polyclonal IgG enhanced invasion in a complement-dependent manner [48].

Apical membrane antigen 1 (AMA1) is a micronemal protein that is transferred to the merozoite surface just prior to merozoite egress [49]. Like MSP1, AMA1 undergoes proteolytic processing [50] to be functional in erythrocyte invasion. The ability of AMA1-specific invasion inhibitory antibodies raised in rabbits to inhibit proteolytic processing [51] may suggest that naturally acquired AMA1-specific antibodies in humans could also function through inhibiting proteolytic processing. Indeed, protective antibodies directed against AMA1 have been reported in children and adults living in some endemic areas [52,53], but these antibodies could be strain-specific due to high polymorphisms seen in AMA1 [54]. Erythrocyte binding antigens (EBAs), especially EBA175, EBA140, and EBA 180, have repeatedly been shown to elicit antibodies, which may possess invasion inhibitory activities in endemic populations [14,45] and they have also been associated with protection. The reticulocyte-binding homologue family of proteins is localized in the rhoptries and is also the target of blood-stage antibody response. Prominent among this protein family are PfRH1, PfRH2, PfRH3, PfRH4, and PfRH5; they coordinate with EBA members to make erythrocyte invasion successful. A study in a Kenyan population showed that antibodies produced against PfRH2a/b and PfRH4 were acquired in an age-dependent manner [45]. A prospective study conducted in Papua New Guinea showed that IgG subclass responses to PfRH2 were predominantly IgG1 and IgG3 and strongly associated with a reduced risk for symptomatic malaria [55]. This study was important because the entire PfRh2a/b was expressed in eight fragments and all of them elicited an antibody response that showed a significant association with reduced risk for malaria. Another study in a low malaria transmission region of Peru also found that the IgG1 response against PfRh2a/b was significantly higher in asymptomatic individuals, and that the elevated total IgG antibody response against the proteins was positively associated with decreased parasitemia [56]. PfRH5 is a potential vaccine, because it is a highly conserved protein and also plays essential roles in invasion [57]. A recent study reported a low seroprevalence of antibodies against PfRH5 in a complex with Pf113 and CyRPA (cysteine-rich protective antigen) in a Ghanaian population [58], and an earlier study [59] also found a low seroprevalence of PfRH5-specific antibodies in Mali, but these antibodies were found to have parasite inhibitory activities. It is intriguing that naturally acquired IgG antibodies produced against highly polymorphic parasite antigens, such as MSP1 and AMA1, are more prevalent and also have approximately two orders of magnitude higher IgG reactivity than PfRH5-specific antibodies. However, it is known from before that not only the level, but also the function of antibodies could be of importance in the development of natural immunity against malaria [60]; thus, even if the concentration of PfRH5 antibodies might not be high, the antibodies could still be of major significance. The majority of the parasite antigens enumerated above, and those not mentioned such as MSP2, MSP2, MSP3, MSP7, MSP9, MSP10, and GAMA, elicit complement fixing antibodies and they can all contribute to the immune system armament against the asexual stage of *P. falciparum* [61]. Protection from an antibody response to a single antigen is not likely to be sufficient for sustained protection against malaria in endemic regions. This was demonstrated in a Kenyan population where high levels of antibodies as well as responses to multiple antigens were found to be predictive of protection against malaria [62].

Interethnic differences in antibody responses to some blood-stage antigens such as ring-infected erythrocyte surface antigen (RESA) and Pf322 have been used as indirect evidence for the existence of an association between naturally acquired immunity and human genetic factors [63]. A genome-wide association that assayed 174950 single nucleotide polymorphisms (SNPs) found 25 SNPs with a possible influence on MSP1 antibody responses [64]. However, an earlier multicenter study in Africa and Asia that assayed 202 SNPs only found moderate evidence for an association of antibody responses to MSP2 and CD36 [65]. Meanwhile, earlier investigations based on classical twin and parent–offspring studies have demonstrated heritability of antibody responses against RESA, MSP1, and MSP2 [66,67,68]. These genetics studies have shown that host factors could also play important roles, apart from parasite factors, in the pattern of natural antibody response observed in endemic regions. Bigger studies involving many different populations and SNPs are needed to show if and how genes have been selected during our co-evolution with *P. falciparum.*

## 4. Antibodies against the Surface of the Infected Red Blood Cell

Obstructions of small blood vessels are caused by binding of proteins expressed by *P. falciparum* on the surface of infected red blood cells (iRBC) to human endothelial cells. This process is central for the development of severe forms of malaria mainly in children. One of the main iRBC proteins is *P. falciparum* erythrocyte membrane protein 1(PfEMP1), and antibodies targeting PfEMP1 have been shown to be associated with a reduced risk of severe malaria in children in endemic areas [69]. A study comparing antibodies against merozoite surface proteins and anti-PfEMP1 antibodies found the latter to be more protective in younger and older children, while the former exerted protective effects only in older children [70]. VAR2CSA is a member of the PfEMP1 protein family that is responsible for sequestration of *P. falciparum*-iRBC in the placenta by binding to chondroitin sulphate A (CSA) [71]. The sequestration of parasites expressing VAR2CSA is mainly responsible for pregnancy-associated malaria (PAM) and causes significant damage to maternal and neonate health in endemic areas. VAR2CSA is produced as a protein with six Duffy-binding like (DBL) domains and three antigenically important interdomain (ID) regions [72]. Natural antibodies targeting these domains and antibodies that prevent CSA binding have been shown to be acquired in a sex and parity-dependent manner in endemic areas and are considered to be protective against PAM [73,74,75,76,77], but they have also been shown to exist in men and children [78]. The main mechanism of action of these antibodies has been shown to be inhibition of binding to CSA, but more cytophilic antibodies directed against VAR2CSA could also function by opsonic phagocytosis of placenta-specific parasites [79,80]. However, a recent systemic and meta-analysis of 33 studies conducted in endemic areas [81] could not find anti-VAR2CSA to be associated with protection against PAM or birth outcomes, but rather with exposure to infection. This strong correlation of anti-VAR2CSA antibodies with exposure has made these antibodies be considered as a powerful tool for monitoring the levels of malaria burden and transmission in endemic areas, irrespective of whether they are protective or not [82,83].

## 5. Autoantibodies at the Blood-Stage

Autoantibodies targeting different human cellular components have been associated with *P. falciparum* infections in endemic areas. Autoantibodies can be directed against membrane phospholipids [84,85,86], erythrocyte membrane proteins [87,88], or DNA [89]. During an acute episode of malaria, it is quite common to become DAT (Direct Antiglobulin Test)-positive, a sign that there are autoantibodies bound to RBC. This can later disappear, even though studies have found around 5% of an apparently healthy adult population in Kenya and Thailand to be DAT-positive [90,91]. Another study has shown a relationship between DAT-positivity and acquired protective immune responses against malaria [92], indicating that these antibodies could have a protective effect.

Not much is known about the mechanisms through which autoantibodies are generated in malaria infection, but it has been suggested that the expression of parasite proteins such as PfEMP1 on erythrocyte membranes could be an initiation step. For instance, a genome-wide study [93] found that PfEMP1 shares a 14-amino acid motif with a human serum protein, vitronectin. This creates a molecular mimicry pathway for autoantibody generation during a normal immune response directed against PfEMP1. Cysteine-rich interdomain region 1 (CIDR1α) of PfEMP1 has also been suggested as a polyclonal activator of B cells that can cause production of non-specific IgM [94], which could be a pathway for autoreactivity. Autoantibodies could also be generated against components such as phosphatidylserine (PS), which is a membrane phospholipid not normally exposed, since it is a resident of the plasma membrane inner leaflet, but it is exposed when the erythrocyte ruptures.

Autoantibodies targeting DNA and other cytoplasmic components could be a form of homeostatic response to mop up cellular fragments, or debris produced during erythrocyte rupture that accompany blood-stage infection, or are generated by oxidative damage associated with antimalarial drug use [95]. If this is the case, then these antibodies will not have any protective effect against future *P. falciparum* infections. On the contrary, they could instead do more harm than good. Autoantibodies have been shown to aggravate malaria pathology by inducing cell lysis, microthrombosis, and inflammation [96]. A recent study demonstrated a clear correlation between kidney injury and autoantibodies against DNA and PS in Ugandan children with severe *P. falciparum* malaria [89]. Anemia, a major symptom of malaria, has also been shown to be associated with anti-PS antibodies [85,89,97]. However, there are also autoantibodies that could be associated with protection against malaria, as found in a Liberian population where antibodies targeting Band 3 neoantigens were found [98], and sera obtained from autoimmune patients could have similar effects [99]. Anti-Band 3 (neoantigen) antibodies correlated with lower parasite density and higher hematocrit [98,100], suggesting a possible role in protection against malaria, even though the correlation could also just be a result of exposure, since the neoantigens are not naturally exposed when there is no malaria. However, it is interesting to note that during the recent years, it has been speculated that having malaria is protective against certain autoimmune diseases such as systemic lupus erythematosus (SLE), and sera of patients with SLE has been shown to inhibit in vitro the growth of *P. falciparum* [101].

## 6. Antibodies at the Human–Mosquito Junction

Gametocytes are specialized and permanently differentiated forms of *Plasmodium* that are responsible for parasite transmission from the human host to the Anopheles mosquito. Mosquitoes pick up gametocytes when they take blood meals from infected humans. In response to the environment in the mosquito lumen, gametocytes transform and after fertilization, they form a zygote. Pfs230 and Pfs48/45 belong to the 6-cystein protein family that is expressed on the surface of gametocytes and play important roles in fertilization [102]. Studies have found naturally produced antibodies against these antigens in most endemic areas, even in individuals with limited exposure to malaria [103]. A more recent study found non-febrile school children living in a high transmission area to produce more anti-Pfs230 antibodies compared with a similar group of children in a low transmission area [104]. Mosquitoes also ingest these antibodies as they take blood meals and the antibodies can exert their transmission blocking activities by blocking fertilization, which is an important step in malaria transmission. Pfs47 is also a member of the 6-cysteine family of proteins [105]. The immune system of *Anopheles* mosquitoes can limit *Plasmodium* infection, but Pfs47 can be used by the parasite to subvert the complement-like immune mechanism of the mosquito in order to establish an infection [106]. The interaction of Pfs47 with its receptor on midgut cells disrupts an apoptosis pathway mediated by the JNK/caspase complex, a step necessary for killing the invading parasite by membrane lysis [107]. The midgut cell receptor of Pfs47 was recently shown to be P47Rec, a highly conserved protein [108]. This efficient evasion of the cellular and humoral components of the mosquito immune system is the first significant barrier cleared by *P. falciparum* parasites in their transmission cycle. It could be challenging to produce effective antibodies against Pfs47 as the majority of monoclonal antibodies produced against full-length recombinant Pfs47 lacked transmission blocking activities, and some antibodies even seemed to increase the transmission [109].

## 7. Conclusions

There is no lack of antibody attacks against *P. falciparum* in the human host as antibodies accompany the parasites through all the stages of development, and human anti-parasite antibodies are even carried on into the mosquitoes. Although the functional roles of autoantibodies are still enigmatic, their contribution to malaria pathogenesis cannot be disregarded. The ability of malaria parasites to complete their life cycle despite the abundance of antibody attacks is probably a high level of evolutionary success with a win–win situation between the human host and the parasites. Antibody responses are able to protect the host enough for the host to survive, but not enough to kill the parasites; thus, parasites can survive in low numbers and be transmitted to a new host. The field of malaria research where combinations of antibodies with different specificities are used together needs more focus in future studies to elucidate what happens in real life in a human being during the development of natural immunity against malaria.

## Data Availability

Not applicable.

## References

[1] World Health Organization (2019). Malaria World Report.

[2] Weiss D.J., Bertozzi-Villa A., Rumisha S.F., Amratia P., Arambepola R., Battle K.E., Cameron E., Chestnutt E., Gibson H.S., Harris J. (2021). Indirect effects of the COVID-19 pandemic on malaria intervention coverage, morbidity, and mortality in Africa: A geospatial modelling analysis. Lancet Infect. Dis..

[3] Deng Z., Wang X., Liu Y., Tian X., Deng S., Sun Y., Wang S., Zheng D., Cui Z., Pan Y. (2020). Single-cell RNA sequencing confirms IgG transcription and limited diversity of VHDJH rearrangements in proximal tubular epithelial cells. Sci. Rep..

[4] Li J., Korteweg C., Qiu Y., Luo J., Chen Z., Huang G., Li W., Gu J. (2014). Two ultrastructural distribution patterns of immunoglobulin G in human placenta and functional implications. Biol. Reprod..

[5] Niu N., Zhang J., Sun Y., Wang S., Sun Y., Korteweg C., Gao W., Gu J. (2011). Expression and distribution of immunoglobulin G and its receptors in an immune privileged site: The eye. Cell Mol. Life Sci..

[6] Qiu X., Zhu X., Zhang L., Mao Y., Zhang J., Hao P., Li G., Lv P., Li Z., Sun X. (2003). Human epithelial cancers secrete immunoglobulin g with unidentified specificity to promote growth and survival of tumor cells. Cancer Res..

[7] Lu L.L., Suscovich T.J., Fortune S.M., Alter G. (2018). Beyond binding: Antibody effector functions in infectious diseases. Nat. Rev. Immunol..

[8] Cohen S., Mc G.I., Carrington S. (1961). Gamma-globulin and acquired immunity to human malaria. Nature.

[9] Edozien J.C., Gilles H.M., Udeozo I.O.K. (1962). Adult and Cord-blood Gamma-globulin and Immunity to Malaria in Nigerians. Lancet.

[10] Kisalu N.K., Idris A.H., Weidle C., Flores-Garcia Y., Flynn B.J., Sack B.K., Murphy S., Schon A., Freire E., Francica J.R. (2018). A human monoclonal antibody prevents malaria infection by targeting a new site of vulnerability on the parasite. Nat. Med..

[11] Amanna I.J., Carlson N.E., Slifka M.K. (2007). Duration of humoral immunity to common viral and vaccine antigens. N. Engl. J. Med..

[12] Akpogheneta O.J., Duah N.O., Tetteh K.K., Dunyo S., Lanar D.E., Pinder M., Conway D.J. (2008). Duration of naturally acquired antibody responses to blood-stage Plasmodium falciparum is age dependent and antigen specific. Infect. Immun..

[13] Crompton P.D., Kayala M.A., Traore B., Kayentao K., Ongoiba A., Weiss G.E., Molina D.M., Burk C.R., Waisberg M., Jasinskas A. (2010). A prospective analysis of the Ab response to Plasmodium falciparum before and after a malaria season by protein microarray. Proc. Natl. Acad. Sci. USA.

[14] Tijani M.K., Babalola O.A., Odaibo A.B., Anumudu C.I., Asinobi A.O., Morenikeji O.A., Asuzu M.C., Langer C., Reiling L., Beeson J.G. (2017). Acquisition, maintenance and adaptation of invasion inhibitory antibodies against Plasmodium falciparum invasion ligands involved in immune evasion. PLoS ONE.

[15] Lugaajju A., Reddy S.B., Wahlgren M., Kironde F., Persson K.E.M. (2017). Development of Plasmodium falciparum specific naive, atypical, memory and plasma B cells during infancy and in adults in an endemic area. Malar. J..

[16] Weiss G.E., Crompton P.D., Li S., Walsh L.A., Moir S., Traore B., Kayentao K., Ongoiba A., Doumbo O.K., Pierce S.K. (2009). Atypical memory B cells are greatly expanded in individuals living in a malaria-endemic area. J. Immunol..

[17] Muellenbeck M.F., Ueberheide B., Amulic B., Epp A., Fenyo D., Busse C.E., Esen M., Theisen M., Mordmuller B., Wardemann H. (2013). Atypical and classical memory B cells produce Plasmodium falciparum neutralizing antibodies. J. Exp. Med..

[18] Gueirard P., Tavares J., Thiberge S., Bernex F., Ishino T., Milon G., Franke-Fayard B., Janse C.J., Menard R., Amino R. (2010). Development of the malaria parasite in the skin of the mammalian host. Proc. Natl. Acad. Sci. USA.

[19] Winkel B.M.F., de Korne C.M., van Oosterom M.N., Staphorst D., Bunschoten A., Langenberg M.C.C., Chevalley-Maurel S.C., Janse C.J., Franke-Fayard B., van Leeuwen F.W.B. (2019). A tracer-based method enables tracking of Plasmodium falciparum malaria parasites during human skin infection. Theranostics.

[20] Winkel B.M.F., de Korne C.M., van Oosterom M.N., Staphorst D., Meijhuis M., Baalbergen E., Ganesh M.S., Dechering K.J., Vos M.W., Chevalley-Maurel S.C. (2019). Quantification of wild-type and radiation attenuated Plasmodium falciparum sporozoite motility in human skin. Sci. Rep..

[21] Hellmann J.K., Munter S., Kudryashev M., Schulz S., Heiss K., Muller A.K., Matuschewski K., Spatz J.P., Schwarz U.S., Frischknecht F. (2011). Environmental constraints guide migration of malaria parasites during transmission. PLoS Pathog..

[22] Hopp C.S., Chiou K., Ragheb D.R., Salman A.M., Khan S.M., Liu A.J., Sinnis P. (2015). Longitudinal analysis of Plasmodium sporozoite motility in the dermis reveals component of blood vessel recognition. Elife.

[23] Flores-Garcia Y., Nasir G., Hopp C.S., Munoz C., Balaban A.E., Zavala F., Sinnis P. (2018). Antibody-Mediated Protection against Plasmodium Sporozoites Begins at the Dermal Inoculation Site. mBio.

[24] Druilhe P., Pradier O., Marc J.P., Miltgen F., Mazier D., Parent G. (1986). Levels of antibodies to Plasmodium falciparum sporozoite surface antigens reflect malaria transmission rates and are persistent in the absence of reinfection. Infect. Immun..

[25] Nardin E.H., Nussenzweig R.S., McGregor I.A., Bryan J.H. (1979). Antibodies to sporozoites: Their frequent occurrence in individuals living in an area of hyperendemic malaria. Science.

[26] Del Giudice G., Engers H.D., Tougne C., Biro S.S., Weiss N., Verdini A.S., Pessi A., Degremont A.A., Freyvogel T.A., Lambert P.H. (1987). Antibodies to the repetitive epitope of Plasmodium falciparum circumsporozoite protein in a rural Tanzanian community: A longitudinal study of 132 children. Am. J. Trop. Med. Hyg..

[27] Hoffman S.L., Wistar R., Ballou W.R., Hollingdale M.R., Wirtz R.A., Schneider I., Marwoto H.A., Hockmeyer W.T. (1986). Immunity to malaria and naturally acquired antibodies to the circumsporozoite protein of Plasmodium falciparum. N. Engl. J. Med..

[28] Marsh K., Hayes R.H., Carson D.C., Otoo L., Shenton F., Byass P., Zavala F., Greenwood B.M. (1988). Anti-sporozoite antibodies and immunity to malaria in a rural Gambian population. Trans. R Soc. Trop. Med. Hyg..

[29] Nwagwu M., Anumudu C.A., Sodeinde O., Ologunde C.A., Obi T.U., Wirtz R.A., Gordon D.M., Lyon J.A. (1998). Identification of a subpopulation of immune Nigerian adult volunteers by antibodies to the circumsporozoite protein of Plasmodium falciparum. Am. J. Trop. Med. Hyg..

[30] Offeddu V., Olotu A., Osier F., Marsh K., Matuschewski K., Thathy V. (2017). High Sporozoite Antibody Titers in Conjunction with Microscopically Detectable Blood Infection Display Signatures of Protection from Clinical Malaria. Front. Immunol..

[31] Shi Y.P., Udhayakumar V., Alpers M.P., Povoa M.M., Oloo A.J., Ruebush T.K., Lal A.A. (1993). Natural antibody responses against the non-repeat-sequence-based B-cell epitopes of the Plasmodium falciparum circumsporozoite protein. Infect. Immun..

[32] Kurtovic L., Behet M.C., Feng G., Reiling L., Chelimo K., Dent A.E., Mueller I., Kazura J.W., Sauerwein R.W., Fowkes F.J.I. (2018). Human antibodies activate complement against Plasmodium falciparum sporozoites, and are associated with protection against malaria in children. BMC Med..

[33] Fowkes F.J., Richards J.S., Simpson J.A., Beeson J.G. (2010). The relationship between anti-merozoite antibodies and incidence of Plasmodium falciparum malaria: A systematic review and meta-analysis. PLoS Med..

[34] Lin C.S., Uboldi A.D., Epp C., Bujard H., Tsuboi T., Czabotar P.E., Cowman A.F. (2016). Multiple Plasmodium falciparum Merozoite Surface Protein 1 Complexes Mediate Merozoite Binding to Human Erythrocytes. J. Biol. Chem..

[35] Bowman N.M., Juliano J.J., Snider C.J., Kharabora O., Meshnick S.R., Vulule J., John C.C., Moormann A.M. (2016). Longevity of Genotype-Specific Immune Responses to Plasmodium falciparum Merozoite Surface Protein 1 in Kenyan Children from Regions of Different Malaria Transmission Intensity. Am. J. Trop. Med. Hyg..

[36] Boyle M.J., Reiling L., Feng G., Langer C., Osier F.H., Aspeling-Jones H., Cheng Y.S., Stubbs J., Tetteh K.K., Conway D.J. (2015). Human antibodies fix complement to inhibit Plasmodium falciparum invasion of erythrocytes and are associated with protection against malaria. Immunity.

[37] Galamo C.D., Jafarshad A., Blanc C., Druilhe P. (2009). Anti-MSP1 block 2 antibodies are effective at parasite killing in an allele-specific manner by monocyte-mediated antibody-dependent cellular inhibition. J. Infect. Dis..

[38] Woehlbier U., Epp C., Hackett F., Blackman M.J., Bujard H. (2010). Antibodies against multiple merozoite surface antigens of the human malaria parasite Plasmodium falciparum inhibit parasite maturation and red blood cell invasion. Malar. J..

[39] Blank A., Fürle K., Jäschke A., Mikus G., Lehmann M., Hüsing J., Heiss K., Giese T., Carter D., Böhnlein E. (2020). Immunization with full-length Plasmodium falciparum merozoite surface protein 1 is safe and elicits functional cytophilic antibodies in a randomized first-in-human trial. NPJ Vaccines.

[40] Das S., Hertrich N., Perrin A.J., Withers-Martinez C., Collins C.R., Jones M.L., Watermeyer J.M., Fobes E.T., Martin S.R., Saibil H.R. (2015). Processing of Plasmodium falciparum Merozoite Surface Protein MSP1 Activates a Spectrin-Binding Function Enabling Parasite Egress from RBCs. Cell Host Microbe.

[41] Nwuba R.I., Sodeinde O., Anumudu C.I., Omosun Y.O., Odaibo A.B., Holder A.A., Nwagwu M. (2002). The human immune response to Plasmodium falciparum includes both antibodies that inhibit merozoite surface protein 1 secondary processing and blocking antibodies. Infect. Immun..

[42] Rono J., Färnert A., Olsson D., Osier F., Rooth I., Persson K.E.M. (2012). Plasmodium falciparum line-dependent association of in vitro growth-inhibitory activity and risk of malaria. Infect. Immun..

[43] Bowyer P.W., Stewart L.B., Aspeling-Jones H., Mensah-Brown H.E., Ahouidi A.D., Amambua-Ngwa A., Awandare G.A., Conway D.J. (2015). Variation in Plasmodium falciparum erythrocyte invasion phenotypes and merozoite ligand gene expression across different populations in areas of malaria endemicity. Infect. Immun..

[44] Gomez-Escobar N., Amambua-Ngwa A., Walther M., Okebe J., Ebonyi A., Conway D.J. (2010). Erythrocyte invasion and merozoite ligand gene expression in severe and mild Plasmodium falciparum malaria. J. Infect. Dis..

[45] Persson K.E.M., McCallum F.J., Reiling L., Lister N.A., Stubbs J., Cowman A.F., Marsh K., Beeson J.G. (2008). Variation in use of erythrocyte invasion pathways by Plasmodium falciparum mediates evasion of human inhibitory antibodies. J. Clin. Investig..

[46] Persson K.E.M., Lee C.T., Marsh K., Beeson J.G. (2006). Development and optimization of high-throughput methods to measure Plasmodium falciparum-specific growth inhibitory antibodies. J. Clin. Microbiol..

[47] Halstead S.B., Mahalingam S., Marovich M.A., Ubol S., Mosser D.M. (2010). Intrinsic antibody-dependent enhancement of microbial infection in macrophages: Disease regulation by immune complexes. Lancet Infect. Dis..

[48] Biryukov S., Angov E., Landmesser M.E., Spring M.D., Ockenhouse C.F., Stoute J.A. (2016). Complement and Antibody-mediated Enhancement of Red Blood Cell Invasion and Growth of Malaria Parasites. EBioMedicine.

[49] Bannister L.H., Hopkins J.M., Dluzewski A.R., Margos G., Williams I.T., Blackman M.J., Kocken C.H., Thomas A.W., Mitchell G.H. (2003). Plasmodium falciparum apical membrane antigen 1 (PfAMA-1) is translocated within micronemes along subpellicular microtubules during merozoite development. J. Cell Sci..

[50] Howell S.A., Well I., Fleck S.L., Kettleborough C., Collins C.R., Blackman M.J. (2003). A single malaria merozoite serine protease mediates shedding of multiple surface proteins by juxtamembrane cleavage. J. Biol. Chem..

[51] Dutta S., Haynes J.D., Moch J.K., Barbosa A., Lanar D.E. (2003). Invasion-inhibitory antibodies inhibit proteolytic processing of apical membrane antigen 1 of Plasmodium falciparum merozoites. Proc. Natl. Acad. Sci. USA.

[52] Polley S.D., Mwangi T., Kocken C.H., Thomas A.W., Dutta S., Lanar D.E., Remarque E., Ross A., Williams T.N., Mwambingu G. (2004). Human antibodies to recombinant protein constructs of Plasmodium falciparum Apical Membrane Antigen 1 (AMA1) and their associations with protection from malaria. Vaccine.

[53] Greenhouse B., Ho B., Hubbard A., Njama-Meya D., Narum D.L., Lanar D.E., Dutta S., Rosenthal P.J., Dorsey G., John C.C. (2011). Antibodies to Plasmodium falciparum antigens predict a higher risk of malaria but protection from symptoms once parasitemic. J. Infect. Dis..

[54] Bailey J.A., Berry A.A., Travassos M.A., Ouattara A., Boudova S., Dotsey E.Y., Pike A., Jacob C.G., Adams M., Tan J.C. (2020). Microarray analyses reveal strain-specific antibody responses to Plasmodium falciparum apical membrane antigen 1 variants following natural infection and vaccination. Sci. Rep..

[55] Reiling L., Richards J.S., Fowkes F.J., Barry A.E., Triglia T., Chokejindachai W., Michon P., Tavul L., Siba P.M., Cowman A.F. (2010). Evidence that the erythrocyte invasion ligand PfRh2 is a target of protective immunity against Plasmodium falciparum malaria. J. Immunol..

[56] Villasis E., Lopez-Perez M., Torres K., Gamboa D., Neyra V., Bendezu J., Tricoche N., Lobo C., Vinetz J.M., Lustigman S. (2012). Anti-Plasmodium falciparum invasion ligand antibodies in a low malaria transmission region, Loreto, Peru. Malar. J..

[57] Baum J., Chen L., Healer J., Lopaticki S., Boyle M., Triglia T., Ehlgen F., Ralph S.A., Beeson J.G., Cowman A.F. (2009). Reticulocyte-binding protein homologue 5—An essential adhesin involved in invasion of human erythrocytes by Plasmodium falciparum. Int. J. Parasitol..

[58] Partey F.D., Castberg F.C., Sarbah E.W., Silk S.E., Awandare G.A., Draper S.J., Opoku N., Kweku M., Ofori M.F., Hviid L. (2018). Kinetics of antibody responses to PfRH5-complex antigens in Ghanaian children with Plasmodium falciparum malaria. PLoS ONE.

[59] Tran T.M., Ongoiba A., Coursen J., Crosnier C., Diouf A., Huang C.Y., Li S., Doumbo S., Doumtabe D., Kone Y. (2014). Naturally acquired antibodies specific for Plasmodium falciparum reticulocyte-binding protein homologue 5 inhibit parasite growth and predict protection from malaria. J. Infect. Dis..

[60] Reddy S.B., Anders R.F., Cross N., Mueller I., Senn N., Stanisic D.I., Siba P.M., Wahlgren M., Kironde F., Beeson J.G. (2015). Differences in affinity of monoclonal and naturally acquired polyclonal antibodies against Plasmodium falciparum merozoite antigens. BMC Microbiol..

[61] Reiling L., Boyle M.J., White M.T., Wilson D.W., Feng G., Weaver R., Opi D.H., Persson K.E.M., Richards J.S., Siba P.M. (2019). Targets of complement-fixing antibodies in protective immunity against malaria in children. Nat. Commun..

[62] Osier F.H., Fegan G., Polley S.D., Murungi L., Verra F., Tetteh K.K., Lowe B., Mwangi T., Bull P.C., Thomas A.W. (2008). Breadth and magnitude of antibody responses to multiple Plasmodium falciparum merozoite antigens are associated with protection from clinical malaria. Infect. Immun..

[63] Modiano D., Chiucchiuini A., Petrarca V., Sirima B.S., Luoni G., Perlmann H., Esposito F., Coluzzi M. (1998). Humoral response to Plasmodium falciparum Pf155/ring-infected erythrocyte surface antigen and Pf332 in three sympatric ethnic groups of Burkina Faso. Am. J. Trop. Med. Hyg..

[64] Milet J., Sabbagh A., Migot-Nabias F., Luty A.J., Gaye O., Garcia A., Courtin D. (2016). Genome-wide association study of antibody responses to Plasmodium falciparum candidate vaccine antigens. Genes Immun..

[65] Shelton J.M., Corran P., Risley P., Silva N., Hubbart C., Jeffreys A., Rowlands K., Craik R., Cornelius V., Hensmann M. (2015). Genetic determinants of anti-malarial acquired immunity in a large multi-centre study. Malar. J..

[66] Aucan C., Traore Y., Fumoux F., Rihet P. (2001). Familial correlation of immunoglobulin G subclass responses to Plasmodium falciparum antigens in Burkina Faso. Infect. Immun..

[67] Duah N.O., Weiss H.A., Jepson A., Tetteh K.K., Whittle H.C., Conway D.J. (2009). Heritability of antibody isotype and subclass responses to Plasmodium falciparum antigens. PLoS ONE.

[68] Sjoberg K., Lepers J.P., Raharimalala L., Larsson A., Olerup O., Marbiah N.T., Troye-Blomberg M., Perlmann P. (1992). Genetic regulation of human anti-malarial antibodies in twins. Proc. Natl. Acad. Sci. USA.

[69] Chan J.A., Boyle M.J., Moore K.A., Reiling L., Lin Z., Hasang W., Avril M., Manning L., Mueller I., Laman M. (2019). Antibody Targets on the Surface of Plasmodium falciparum-Infected Erythrocytes That Are Associated With Immunity to Severe Malaria in Young Children. J. Infect. Dis..

[70] Chan J.A., Stanisic D.I., Duffy M.F., Robinson L.J., Lin E., Kazura J.W., King C.L., Siba P.M., Fowkes F.J., Mueller I. (2017). Patterns of protective associations differ for antibodies to P. falciparum-infected erythrocytes and merozoites in immunity against malaria in children. Eur. J. Immunol..

[71] Salanti A., Staalsoe T., Lavstsen T., Jensen A.T., Sowa M.P., Arnot D.E., Hviid L., Theander T.G. (2003). Selective upregulation of a single distinctly structured var gene in chondroitin sulphate A-adhering Plasmodium falciparum involved in pregnancy-associated malaria. Mol. Microbiol..

[72] Clausen T.M., Christoffersen S., Dahlback M., Langkilde A.E., Jensen K.E., Resende M., Agerbaek M.O., Andersen D., Berisha B., Ditlev S.B. (2012). Structural and functional insight into how the Plasmodium falciparum VAR2CSA protein mediates binding to chondroitin sulfate A in placental malaria. J. Biol. Chem..

[73] Brolin K.J., Persson K.E.M., Wahlgren M., Rogerson S.J., Chen Q. (2010). Differential recognition of P. falciparum VAR2CSA domains by naturally acquired antibodies in pregnant women from a malaria endemic area. PLoS ONE.

[74] Fried M., Nosten F., Brockman A., Brabin B.J., Duffy P.E. (1998). Maternal antibodies block malaria. Nature.

[75] O’Neil-Dunne I., Achur R.N., Agbor-Enoh S.T., Valiyaveettil M., Naik R.S., Ockenhouse C.F., Zhou A., Megnekou R., Leke R., Taylor D.W. (2001). Gravidity-dependent production of antibodies that inhibit binding of Plasmodium falciparum-infected erythrocytes to placental chondroitin sulfate proteoglycan during pregnancy. Infect. Immun..

[76] Ricke C.H., Staalsoe T., Koram K., Akanmori B.D., Riley E.M., Theander T.G., Hviid L. (2000). Plasma antibodies from malaria-exposed pregnant women recognize variant surface antigens on Plasmodium falciparum-infected erythrocytes in a parity-dependent manner and block parasite adhesion to chondroitin sulfate A. J. Immunol..

[77] Salanti A., Dahlbäck M., Turner L., Nielsen M.A., Barfod L., Magistrado P., Jensen A.T., Lavstsen T., Ofori M.F., Marsh K. (2004). Evidence for the involvement of VAR2CSA in pregnancy-associated malaria. J. Exp. Med..

[78] Beeson J.G., Ndungu F., Persson K.E.M., Chesson J.M., Kelly G.L., Uyoga S., Hallamore S.L., Williams T.N., Reeder J.C., Brown G.V. (2007). Antibodies among men and children to placental-binding Plasmodium falciparum-infected erythrocytes that express var2csa. Am. J. Trop. Med. Hyg..

[79] Elliott S.R., Brennan A.K., Beeson J.G., Tadesse E., Molyneux M.E., Brown G.V., Rogerson S.J. (2005). Placental malaria induces variant-specific antibodies of the cytophilic subtypes immunoglobulin G1 (IgG1) and IgG3 that correlate with adhesion inhibitory activity. Infect. Immun..

[80] Lambert L.H., Bullock J.L., Cook S.T., Miura K., Garboczi D.N., Diakite M., Fairhurst R.M., Singh K., Long C.A. (2014). Antigen reversal identifies targets of opsonizing IgGs against pregnancy-associated malaria. Infect. Immun..

[81] Cutts J.C., Agius P.A., Zaw L., Powell R., Moore K., Draper B., Simpson J.A., Fowkes F.J.I. (2020). Pregnancy-specific malarial immunity and risk of malaria in pregnancy and adverse birth outcomes: A systematic review. BMC Med..

[82] Ataide R., Mayor A., Rogerson S.J. (2014). Malaria, primigravidae, and antibodies: Knowledge gained and future perspectives. Trends Parasitol..

[83] Fonseca A.M., Gonzalez R., Bardaji A., Jairoce C., Ruperez M., Jimenez A., Quinto L., Cistero P., Vala A., Sacoor C. (2019). VAR2CSA Serology to Detect Plasmodium falciparum Transmission Patterns in Pregnancy. Emerg. Infect. Dis..

[84] Adebajo A.O., Charles P., Maini R.N., Hazleman B.L. (1993). Autoantibodies in malaria, tuberculosis and hepatitis B in a west African population. Clin. Exp. Immunol..

[85] Fernandez-Arias C., Rivera-Correa J., Gallego-Delgado J., Rudlaff R., Fernandez C., Roussel C., Gotz A., Gonzalez S., Mohanty A., Mohanty S. (2016). Anti-Self Phosphatidylserine Antibodies Recognize Uninfected Erythrocytes Promoting Malarial Anemia. Cell Host Microbe.

[86] Rivera-Correa J., Mackroth M.S., Jacobs T., Schulze Zur Wiesch J., Rolling T., Rodriguez A. (2019). Atypical memory B-cells are associated with Plasmodium falciparum anemia through anti-phosphatidylserine antibodies. Elife.

[87] Berzins K., Wahlgren M., Perlmann P. (1983). Studies on the specificity of anti-erythrocyte antibodies in the serum of patients with malaria. Clin. Exp. Immunol..

[88] Fontaine A., Pophillat M., Bourdon S., Villard C., Belghazi M., Fourquet P., Durand C., Lefranc D., Rogier C., Fusai T. (2010). Specific antibody responses against membrane proteins of erythrocytes infected by Plasmodium falciparum of individuals briefly exposed to malaria. Malar. J..

[89] Rivera-Correa J., Conroy A.L., Opoka R.O., Batte A., Namazzi R., Ouma B., Bangirana P., Idro R., Schwaderer A.L., John C.C. (2019). Autoantibody levels are associated with acute kidney injury, anemia and post-discharge morbidity and mortality in Ugandan children with severe malaria. Sci. Rep..

[90] Abdalla S.H., Kasili F.G., Weatherall D.J. (1983). The coombs direct antiglobulin test in Kenyans. Trans. R. Soc. Trop. Med. Hyg..

[91] Merry A.H., Looareesuwan S., Phillips R.E., Chanthavanich P., Supanaranond W., Warrell D.A., Weatherall D.J. (1986). Evidence against immune haemolysis in falciparum malaria in Thailand. Br. J. Haematol..

[92] Abdalla S., Weatherall D.J. (1982). The direct antiglobulin test in P. falciparum malaria. Br. J. Haematol..

[93] Ludin P., Nilsson D., Maser P. (2011). Genome-wide identification of molecular mimicry candidates in parasites. PLoS ONE.

[94] Donati D., Zhang L.P., Chene A., Chen Q., Flick K., Nyström M., Wahlgren M., Bejarano M.T. (2004). Identification of a polyclonal B-cell activator in Plasmodium falciparum. Infect. Immun..

[95] Kavishe R.A., Koenderink J.B., Alifrangis M. (2017). Oxidative stress in malaria and artemisinin combination therapy: Pros and Cons. FEBS J..

[96] Ludwig R.J., Vanhoorelbeke K., Leypoldt F., Kaya Z., Bieber K., McLachlan S.M., Komorowski L., Luo J., Cabral-Marques O., Hammers C.M. (2017). Mechanisms of Autoantibody-Induced Pathology. Front. Immunol..

[97] Rivera-Correa J., Rodriguez A. (2020). Autoimmune Anemia in Malaria. Trends Parasitol..

[98] Hogh B., Petersen E., Crandall I., Gottschau A., Sherman I.W. (1994). Immune responses to band 3 neoantigens on Plasmodium falciparum-infected erythrocytes in subjects living in an area of intense malaria transmission are associated with low parasite density and high hematocrit value. Infect. Immun..

[99] Brahimi K., Martins Y.C., Zanini G.M., Ferreira-da-Cruz Mde F., Daniel-Ribeiro C.T. (2011). Monoclonal auto-antibodies and sera of autoimmune patients react with Plasmodium falciparum and inhibit it’s in vitro growth. Mem Inst. Oswaldo Cruz.

[100] Pantaleo A., Giribaldi G., Mannu F., Arese P., Turrini F. (2008). Naturally occurring anti-band 3 antibodies and red blood cell removal under physiological and pathological conditions. Autoimmun. Rev..

[101] Zanini G.M., De Moura Carvalho L.J., Brahimi K., De Souza-Passos L.F., Guimaraes S.J., Da Silva Machado E., Bianco-Junior C., Riccio E.K., De Sousa M.A., Alecrim M.D. (2009). Sera of patients with systemic lupus erythematosus react with plasmodial antigens and can inhibit the in vitro growth of Plasmodium falciparum. Autoimmunity.

[102] Pradel G. (2007). Proteins of the malaria parasite sexual stages: Expression, function and potential for transmission blocking strategies. Parasitology.

[103] Bousema J.T., Drakeley C.J., Kihonda J., Hendriks J.C., Akim N.I., Roeffen W., Sauerwein R.W. (2007). A longitudinal study of immune responses to Plasmodium falciparum sexual stage antigens in Tanzanian adults. Parasite Immunol..

[104] Amoah L.E., Abagna H.B., Ayanful-Torgby R., Blankson S.O., Aryee N.A. (2019). Diversity and immune responses against Plasmodium falciparum gametocytes in non-febrile school children living in Southern Ghana. Malar. J..

[105] Molina-Cruz A., Canepa G.E., Barillas-Mury C. (2017). Plasmodium P47: A key gene for malaria transmission by mosquito vectors. Curr. Opin. Microbiol..

[106] Molina-Cruz A., Garver L.S., Alabaster A., Bangiolo L., Haile A., Winikor J., Ortega C., van Schaijk B.C., Sauerwein R.W., Taylor-Salmon E. (2013). The human malaria parasite Pfs47 gene mediates evasion of the mosquito immune system. Science.

[107] Ramphul U.N., Garver L.S., Molina-Cruz A., Canepa G.E., Barillas-Mury C. (2015). Plasmodium falciparum evades mosquito immunity by disrupting JNK-mediated apoptosis of invaded midgut cells. Proc. Natl. Acad. Sci. USA.

[108] Molina-Cruz A., Canepa G.E., Alves E.S.T.L., Williams A.E., Nagyal S., Yenkoidiok-Douti L., Nagata B.M., Calvo E., Andersen J., Boulanger M.J. (2020). Plasmodium falciparum evades immunity of anopheline mosquitoes by interacting with a Pfs47 midgut receptor. Proc. Natl. Acad. Sci. USA.

[109] Canepa G.E., Molina-Cruz A., Yenkoidiok-Douti L., Calvo E., Williams A.E., Burkhardt M., Peng F., Narum D., Boulanger M.J., Valenzuela J.G. (2018). Antibody targeting of a specific region of Pfs47 blocks Plasmodium falciparum malaria transmission. NPJ Vaccines.

